# Empirical tests of habitat selection theory reveal that conspecific density and patch quality, but not habitat amount, drive long‐distance immigration in a wild bird

**DOI:** 10.1111/ele.13729

**Published:** 2021-03-20

**Authors:** Clark S. Rushing, T. Brandt Ryder, Jonathon J. Valente, T. Scott Sillett, Peter P. Marra

**Affiliations:** ^1^ Department of Wildland Resources and the Ecology Center Utah State University 5230 Old Main Hill Logan UT 84322 USA; ^2^ Smithsonian Migratory Bird Center National Zoological Park Washington DC 20013 USA; ^3^ Bird Conservancy of the Rockies Fort Collins CO 80525 USA; ^4^ Department of Forest Engineering, Resources, and Management Oregon State University Corvallis OR 97331 USA; ^5^ Department of Biology and McCourt School of Public Policy Georgetown University 37th and O Streets, NW Washington DC 20057 USA

**Keywords:** Conspecific attraction, deuterium, habitat selection, ideal free distribution, immigration, social cues hypothesis, source‐sink dynamics

## Abstract

Individuals that disperse long distances from their natal site must select breeding patches with no prior knowledge of patch suitability. Despite decades of theoretical studies examining which cues dispersing individuals should use to select breeding patches, few empirical studies have tested the predictions of these theories at spatial scales relevant to long‐distance dispersal in wild animal populations. Here, we use a novel assignment model based on multiple intrinsic markers to quantify natal dispersal distances of Wood Thrush (*Hylocichla mustelina*) breeding in forest fragments. We show that long‐distance natal dispersal in this species is more frequent than commonly assumed for songbirds and that habitat selection by these individuals is driven by density‐dependence and patch quality but not the amount of habitat surrounding breeding patches. These results represent an important contribution to understanding habitat selection by dispersing individuals, especially with regards to long‐distance dispersal.

## Graphical abstract

Long‐distance dispersers must select breeding sites with no prior knowledge of site quality, but the cues they use to make these decisions are poorly understood. We used intrinsic markers to show that natal dispersing Wood Thrush favor high‐quality patches but avoid patches with both very low and very high conspecific density. These results suggest that dispersing individuals use both social cues and habitat features when selecting breeding patches.

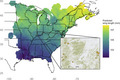

## INTRODUCTION

The ecological factors that influence immigration by dispersing individuals play a central role in the redistribution of individuals and their genes across the landscape. Although passively dispersing organisms have little choice over where they end up, actively dispersing organisms have the ability to select among breeding patches that often differ in key characteristics, including size, quality, and conspecific density. Theoretical studies have established a broad foundation for understanding how these ecological factors influence habitat selection by dispersing individuals (MacArthur & Wilson 2001; Stamps 2001; Bowler & Benton 2005; Clobert *et al.,* 2012; Avgar *et al.,* 2020) and empirical studies have investigated how these factors influence habitat selection in a wide variety of taxa (Morris 1987; Petit & Petit 1996; Jones 2001; Schmidt *et al.,* 2015). However, the relative importance of patch characteristics for habitat selection decisions is expected to vary as a function of dispersal distance (Stamps 2001) due to scale‐dependent costs and benefits of dispersal (Rousset & Gandon 2002; Muller‐Landau *et al.,* 2003). In studies of wild populations, however, the origin of immigrating individuals is rarely known (Millon *et al.,* 2019), precluding inferences about how dispersers select breeding patches. Understanding how patch characteristics and density dependence influence habitat selection is particularly important for long‐distance dispersers because these individuals face unique costs relative to short‐distance dispersers (e.g., increased search costs and mortality or decreased likelihood of locating suitable habitat; Higgins *et al.,* 2003; Muller‐Landau *et al.,* 2003; Stamps *et al.,* 2005) and because rare long‐distance dispersal events can disproportionately influence ecological and evolutionary processes (Kot *et al.,* 1996; Higgins & Richardson 1999; Trakhtenbrot *et al.,* 2005). Empirical studies of habitat selection by long‐distance dispersers are, however, virtually non‐existent (but see Lowe 2009).

**Figure 1:**
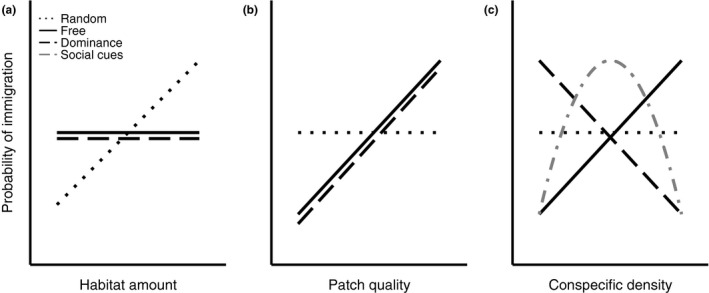
Predictions about the relationship between the probability of long‐distance immigration and (a) habitat amount, (b) patch quality, and (c) conspecific density under four habitat selection hypotheses. See text for descriptions of each hypothesis. Under the social cues hypothesis, the predicted relationship between immigration, habitat amount, and patch quality are the same as under the ideal dominance hypothesis and are therefore not shown. Note that figures show predicted relationships between the probability of long‐distance immigration and each explanatory variable, not absolute differences in immigration rates among the different hypotheses.

Early predictions about habitat selection, derived from island biogeography and metapopulation theory, posited that dispersal was a random process and the probability of immigration was a function of patch isolation and size (Hanski & Hanski 1999; MacArthur & Wilson 2001). Under this model, individuals are ‘free’ in the sense that they are not prevented by conspecifics from settling in whichever patch they locate and the probability of long‐distance immigration is positively related to the availability of habitat (i.e., patch size) but unrelated to patch quality or conspecific density (Boughton 2000). Hereafter, we refer to this as the ‘random’ immigration hypothesis (Fig. 1). Random immigration may be particularly relevant to long‐distance dispersers constrained by search time (Kokko 1999; Smith & Moore 2005) because they have limited prior knowledge of local habitat cues (Part & Gustafsson 1989) and typically cannot directly sample multiple patches prior to breeding (Stamps *et al.,* 2005; Bonte *et al.,* 2012). As a result, long‐distance dispersers may be forced to settle in the first patch they encounter (Stamps 2001; Davis & Stamps 2004).

Many animal species, however, have the ability to search for and colonise patches in a non‐random manner. When dispersal is non‐random, patch quality and conspecific density, rather than habitat amount, are expected to influence habitat selection. Immigrants that can reliably assess habitat and are free to settle should select the highest‐quality patches until density‐dependent mechanisms reduce expected reproductive success to the point where individuals would do better by settling in lower quality patches. This hypothesis corresponds to the ‘ideal free’ distribution (Fretwell & Lucas 1970) and predicts that the probability of long‐distance immigration is positively correlated with habitat quality. Additionally, because the highest‐quality patches are expected to experience the slowest density‐dependent declines in fitness (Avgar *et al.,* 2020), the probability of long‐distance immigration should be positively correlated with conspecific density under this hypothesis (Fig. 1).

When settlement decisions are mediated by intraspecific interactions, the relationships between immigration, patch quality, and conspecific density are more complicated. First, established and/or behaviorally dominant individuals may prevent immigrants from settling in high‐quality patches, in which case immigrants may be forced to settle in low‐quality patches (Fretwell & Lucas 1970; Pulliam 1988). Under this scenario, termed the ‘ideal dominance’ hypothesis (Fretwell & Lucas 1970), immigrants still choose the highest‐quality patch available but dominance hierarchies produce a negative relationship between conspecific density and probability of immigration (Fig. 1). Alternatively, immigrants may rely on conspecific or habitat cues to avoid selecting low‐quality breeding patches. Research on territorial songbirds indicates that individuals preferentially settle in patches occupied by conspecifics (termed conspecific attraction; Stamps 1988; Muller *et al.,* 1997), though actual settlement decisions may be modified by conspecific density (Fletcher 2007), patch quality (Rushing *et al.,* 2015), and the competitive ability or quality of conspecifics (Laland 2004; Szymkowiak *et al.,* 2016). Under this hypothesis, which we refer to as the ‘social cues’ hypothesis, the relationship between immigration and conspecific density is predicted to be non‐linear (high immigration at intermediate densities) because low‐density patches lack conspecific cues and despotism/preemption precludes settlement in high density patches (Fletcher 2007). Although the ‘ideal dominance’ and ‘social cues’ hypotheses make different predictions about the relationship between immigration and density, both scenarios predict that individuals are ideal in their settlement choices. Therefore, after controlling for the effects of density, immigration will be positively related to patch quality (Fig. 1).

The random, ideal free, ideal dominance, and social cues hypotheses make alternative predictions about the relationships between immigration and patch characteristics, but empirical tests of these hypotheses are rare due to the inherent challenges of tracking dispersing individuals over large spatial scales and distinguishing immigrants from local recruits (reviewed by Diffendorfer 1998; Furrer & Pasinelli 2016). Intrinsic markers, including stable isotopes and genetic markers, provide an emerging method for studying long‐distance immigration that can overcome these challenges (López‐Calderón *et al.,* 2019). In particular, because the geographic origin of individuals can be inferred from intrinsic markers without having to track individuals as they disperse, these markers allow researchers to obtain large sample sizes that are not biased towards short‐distance dispersal events and allow inferences to be made about most or all individuals in a study population. Methods that use intrinsic markers can also incorporate multiple sources of information (e.g., abundance, morphology), which can improve the accuracy and resolution of geographic assignments (Rundel *et al.,* 2013; Rushing *et al.,* 2017b). Current methods for studying dispersal using intrinsic markers, however, have limited ability to translate marker values into geographic dispersal distances and quantify uncertainty (López‐Calderón *et al.,* 2019). Analytical approaches that account for sources of error and quantify uncertainty in dispersal distances estimated from intrinsic markers will advance our understanding of the mechanisms that drive immigration and habitat selection.

Here we present a novel analytical framework that estimates dispersal distance from intrinsic markers and test theoretical predictions about the relative importance of habitat amount, patch quality, and conspecific density in determining long‐distance immigration. We used this method to estimate dispersal distance of a migratory songbird, the Wood Thrush (*Hylocichla mustelina*), by combining intrinsic markers, in this case stable hydrogen isotopes and morphological data, with prior information about the distribution of dispersal distances and the relative abundance of source locations. Uncertainty in individual dispersal distances and the probability of long‐distance immigration are estimated through bootstrapping. Our results provide a large‐scale test of immigration theory in a wild animal population and advance our understanding of how ecological factors drive patterns of natal dispersal and breeding settlement. In addition, the methods described in this paper can be readily applied to other types of intrinsic markers (e.g., genetic data or trace elements), thereby advancing the study of immigration and dispersal in other species.

## MATERIALS AND METHODS

### Data

**Figure 2:**
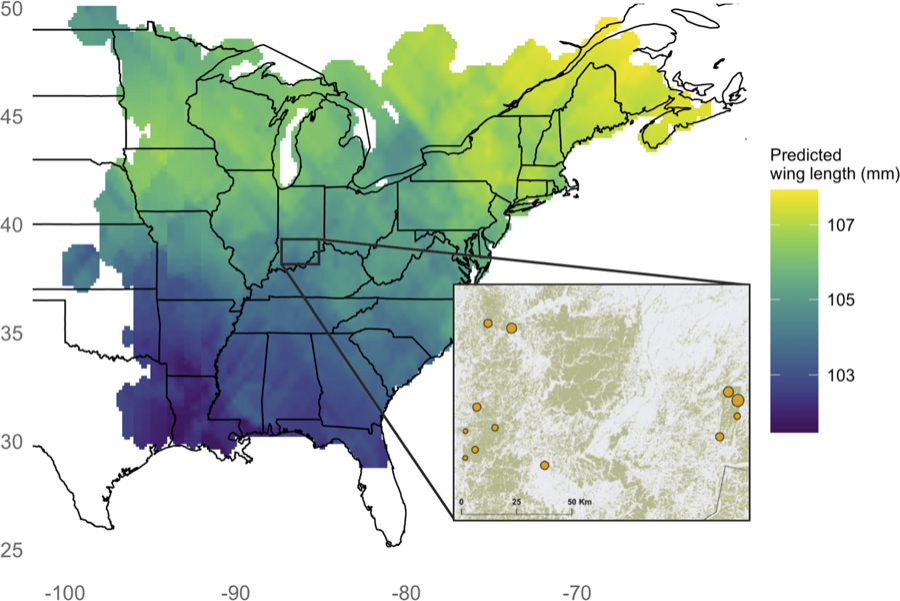
Predicted wing length (mm) of second‐year Wood Thrush across the species' breeding distribution. See text for description of wing length data and kriging process. Inset shows forest cover (green) surrounding the 12 study plots (orange circles) in southern Indiana, USA.

Our geographic assignments of origin are based on stable hydrogen isotope data and morphological measurements collected between 2011 and 2014 from breeding Wood Thrush at 12 study plots in southern Indiana, USA (Fig. 2). Starting in late‐April each year, each plot was systematically surveyed to locate adult Wood Thrush. Territorial adults were captured in mist nets, banded with a USGS aluminum band and a unique combination of colors bands, aged (second‐year or after second‐year), sexed using molt and plumage criteria, measured (unflattened wing chord and tarsus length to ± 0.1 mm), and weighed. In addition, we collected a single tail feather (R3) for stable isotope analysis. Because we expected natal dispersal to be more extensive than breeding dispersal (Greenwood & Harvey 1982; Paradis *et al.,* 1998), only data from individuals in their first breeding season (i.e., aged as second‐year) were included in our analysis (Table 1). We pooled data across years and sexes because we had insufficient sample sizes to estimate annual and sex‐specific patterns of natal dispersal.

**Table 1:** Self‐recruitment rate (R), habitat amount (in hectares), conspecific density (average number of Wood Thrush per hectare), number of second‐year Wood Thrush (n) sampled on each study plot, and the median estimated number of long‐distance immigrants under each of the three disperal kernel priors ($\Gamma_{1}$ = most restrictive, $\Gamma_{3}$ = least restrictive. See text for additional details). Values in parentheses indicate 95% confidence intervals for the number of immigrants

					Estimated number of immigrants		
Plot	*R*	Habitat amount	Density	*n*	$\Gamma_{1}$	$\Gamma_{2}$	$\Gamma_{3}$
1	1.02	1242	1.17	82	2 (0–5)	6 (2–11)	14 (8–21)
2	1.22	1044	0.62	80	3 (0–6)	7 (3–13)	16 (9–23)
3	1.09	885	0.92	33	1 (0–4)	3 (0–7)	7 (3–12)
4	0.94	805	1.13	42	1 (0–4)	3 (1–8)	8 (4–14)
5	0.75	1172	0.50	41	0 (0–2)	1 (0–4)	4 (1–8)
6	0.62	1101	0.43	23	0 (0–2)	1 (0–4)	4 (1–8)
7	0.89	924	0.76	22	0 (0–2)	1 (0–4)	3 (0–6)
8	1.07	929	1.62	39	0 (0–2)	2 (0–5)	4 (1–8)
9	0.77	1139	1.06	35	1 (0–3)	2 (0–5)	4 (1–9)
10	0.60	1112	0.36	34	0 (0–2)	1 (0–4)	4 (1–8)
11	0.88	678	0.88	44	0 (0–2)	2 (0–5)	5 (1–9)
12	0.90	1202	0.52	31	0 (0–2)	1 (0–4)	4 (1–8)

The 12 study plots differed in size, Wood Thrush density, and habitat quality (Table 1). We defined patch quality as the self‐recruitment rate (R) of each plot reported by Rushing *et al.,* (2017a), which was calculated based on the estimated fecundity, juvenile survival, and adult survival of each plot. Self‐recruitment rate is the expected population growth rate of a population in the absence of immigration (Runge *et al.,* 2006); in other words, the ability of a population to maintain itself through only local survival and recruitment. We consider R to be a holistic and direct measure of patch quality, with larger R values indicating higher quality patches for Wood Thrush (Rushing *et al.,* 2017a). To estimate Wood Thrush density we conducted three point counts per breeding season at points spaced in a 200 m grid pattern across the plots. During each count we recorded the distance to all individual Wood Thrush seen or heard and estimated annual plot‐level densities (adjusted for imperfect detection) using a generalised distance sampling model (Chandler *et al.,* 2011). We then calculated a mean annual plot‐level density by averaging the estimates across years. Patch quality and Wood Thrush density were moderately correlated (ρ = 0.53). Quantifying habitat amount is more challenging in part because perceptual range and/or responses may vary in a species‐specific manner (Betts *et al.,* 2014). We quantified habitat amount as the area within a 2 km radius circle centered on each plot that was classified as ‘deciduous forest’ by the National Land Cover Database (NLCD; Fry *et al.,* 2011). We chose 2 km because this scale has been shown to be relevant to habitat use (Mitchell *et al.,* 2001) and extra‐territorial movements (Norris & Stutchbury 2001) in forest breeding songbirds and because, in our study region, this metric is highly correlated with habitat amount measured at scales from 1‐5 km (Valente & Betts 2019). This metric is valid for testing the random immigration hypothesis because the probability of dispersing Wood Thrush randomly encountering potential breeding habitat will increase with increasing forest amount. Habitat amount was not strongly correlated with either patch quality (ρ = −0.27) or Wood Thrush density (ρ = −0.34).

### Spatial variation in intrinsic markers

Stable hydrogen isotope ratios in precipitation ($\delta^{2}H_{p}$) vary along predictable latitudinal and elevational gradients across North America. These gradients are incorporated into animal tissues, providing a chemical fingerprint of the geographic origin of those tissues (Hobson & Wassenaar 1996). Juvenile Wood Thrush molt their tail feathers at their natal site prior to fall migration, therefore stable hydrogen isotope values from feathers ($\delta^{2}H_{f}$) collected at the beginning of one breeding season provide information about an individual's natal origin and dispersal distance (Hobson 2005). We washed, weighed, and analyzed feather samples at the Smithsonian's Stable Isotope Mass Spectrometry Lab using the procedures described by Rushing *et al.,* (2014). Predicted $\delta^{2}H_{f}$ values for 30 × 30 km grid cells covering the known Wood Thrush breeding range were estimated from a published map of expected amount‐weighted growing‐season $\delta^{2}H_{p}$ values (Bowen *et al.,* 2005), converted to the expected feather values using a published correction factor for ground‐foraging migratory birds ($\delta^{2}H_{f}=‐175.57+0.95\delta^{2}H_{p}$; Hobson *et al.,* 2012), and clipped to include only the known Wood Thrush breeding range (Fig. 2).

Wood Thrush also exhibit geographic variation in wing length (denoted W), with individuals from northern populations on average having longer wings than individuals from southern populations (Fig. 2). This variation provides an additional source of information regarding an individual's natal origin and has been successfully incorporated into isotope‐based assignments of non‐breeding Wood Thrush (Rushing *et al.,* 2014). Predicted W for each of the potential natal locations defined by the $\delta^{2}H_{f}$ basemap was estimated by kriging wing chord measurements from 2271 breeding Wood Thrush sampled at as part of the Monitoring Avian Productivity and Survivorship (MAPS) program between 2002 and 2011 (DeSante and Kaschube 2009). See Rushing *et al.,* (2014) for additional details about the kriging process.

### Assignment model

Based on the individual measurements of $\delta^{2}H_{f}$ and W, which we collectively denote y∗, and the basemaps describing spatial variation in the expected value of these markers, the likelihood that a given location j is the natal origin of each individual was defined using a multi‐variate normal likelihood function:

(1) $$\left[y*|\pi_{j},\sum \right]=\frac{\exp(‐\frac{1}{2}{\left(y*‐\pi_{j}\right)}^{T}\sum^{‐1}\left(y*‐\pi_{j}\right))}{\sqrt{{\left(2\pi \right)}^{2}\left|\sum \right|}}$$

where $\mu_{j}$ is the expected $\delta^{2}H_{f}$ and W values for location j and the variance‐covariance matrix Σ describing local variation in the markers (Rushing *et al.,* 2014). We estimated Σ based on the sample variances and covariance of $\delta^{2}H$ and W values at our Indiana study plots:

$$\Sigma =\left[\begin{array}{ccc} 47.88 & ‐1.21 & \\ ‐1.21 & 9.20 \end{array}\right]$$

In addition to the information provided by the $\delta^{2}H_{f}$ and W data, our assignment model takes advantage of prior information about which sites are most likely to be the natal origin of individuals in our study populations. In particular, we expect: (1) sites that are closer to the focal populations are more likely to be origins than distant sites; and (2) sites with larger breeding populations are more likely to be origins than sites with smaller breeding populations. The first expectation stems from the wealth of data indicating that dispersal probability declines with distance in most organisms (Greenwood & Harvey 1982; Paradis *et al.,* 1998; Nathan *et al.,* 2003). This phenomenon can be formalised by defining a dispersal kernel that governs the probability of dispersing between two sites as a function of the distance between sites:

(2) $$\left[D=d_{i,j}|\Gamma \right]=g\left(d,\Gamma \right)$$

where $\left[D=d_{i,j}|\Gamma \right]$ is the likelihood of dispersing distance D, $d_{i,j}$ is the distance between the site i and j, and g is a probability distribution with parameter(s) Γ. Ideally, the distribution g and parameter(s) Γ would be estimated directly from data on natal dispersal distances but this information is currently lacking for Wood Thrush. Most evidence, however, indicates that natal dispersal kernels of songbirds are heavy‐tailed (Paradis *et al.,* 1998), with more frequent long‐distance dispersal events than predicted by a negative exponential distribution. One such kernel is the Weibull distribution, which we used to define g.

The Weibull dispersal kernel is defined by two parameters Γ=[υ,λ], where υ>0and λ>0. Following convention, we refer to υas the *shape* and λas the *scale*. To account for uncertainty in the shape of the Wood Thrush natal dispersal kernel, we conducted our assignments using three kernels that had the same median dispersal distance but imply different levels of long‐distance dispersal. Specifically, we used $\Gamma_{1}=\left[0.75,\;9.26\right]$, $\Gamma_{2}=\left[0.675,\;9.78\right]$, and $\Gamma_{3}=\left[0.60,\;10.47\right]$. These three kernels have a median dispersal distance of 5.7 km, which we estimated using the scaling function estimated by Sutherland *et al.,* (2000) to predict median natal dispersal distance of birds based on body mass (adult Wood Thrush are *c*. 50 g). Given a fixed median dispersal distance, the three υvalues imply increasing frequency of long‐distance dispersal, with $\Gamma_{1}$imposing the most restrictive prior on long‐distance dispersal and $\Gamma_{3}$providing the least restrictive prior. Scale parameters were chosen to ensure the same median dispersal distance in each of the three kernels $(\lambda =5.7/\ln{\left(2\right)}^{1/\upsilon }).$

The second prior expectation, that high abundance sites are more likely origins than low abundance sites, assumes that larger breeding populations produce a larger pool of potential dispersers than sites with small populations. In assignment models, this expectation can be formalised by assuming that the probability that an individual originated from a breeding site is equal to the relative abundance of that site (Royle & Rubenstein 2004):

(3) $$\left[j\right]=N_{j}$$

where $N_{j}$ is the relative abundance of site j and $\sum_{j=1}^{J}N_{j}=1$. We used distribution maps created using count data from the North American Breeding Bird Survey (BBS; Sauer et al. 2015) to create a base map of Wood Thrush breeding abundance (Rushing *et al.,* 2014). Briefly, we predicted abundance in each grid cell as a distance‐weighted average of the observed counts from nearby survey routes (Sauer et al. 2015). We converted the predicted abundance estimates into a probability surface by dividing the abundance of each cell by the total abundance of all cells. Wood Thrush are easily detected during the BBS sampling period and BBS routes cover the entire breeding range, so this map should provide reasonable characterisation of spatial variation in Wood Thrush breeding abundance.

Using Bayes theorem, we combined eqns (1), (2), (4), (5) to estimate the posterior probability that any site j was the origin of an individual with markers y∗:

(4) $$\left[j|y*,\theta_{j},d_{i,j},\Gamma ,N_{j}\right]=\frac{\left[y*|\theta_{j}\right]\left[D=d_{i,j}|\Gamma \right]\left[N_{j}\right]}{\sum_{j=1}^{J}\left[y*|\theta_{j}\right]\left[D=d_{i,j}|\Gamma \right]\left[N_{j}\right]}$$

### Estimation of dispersal distance

The posterior probabilities estimated from Eq. 4 provide a summary of the most likely natal origins for each individual. For our analysis, however, it was necessary to convert these probabilities into a single natal origin to estimate the dispersal distance of each individual. To probabilistically assign each individual k to a single origin location $b_{k}$, we used a categorical distribution defined by the vector $\pi_{k}$ of posterior probabilities for each of the J potential breeding sites:

(5) $$\left[b_{k}|\pi_{k}\right]∼categorical\left(\pi_{k}\right)$$

and dispersal distance $D_{k}$ was defined as the distance between $b_{k}$ and the breeding plot of individual k. To determine the degree to which dispersal distances estimated from the assignment model compare to the distances implied by each prior, we compared the dispersal kernel fitted to our estimates of $D_{k}$ to the prior distributions. Because we chose a Weibull distribution for the prior distributions, we fit a Weibull distribution to the estimated dispersal distances using the R package fitdistrplus (Delignete‐Muller & Dutang 2015). To account for uncertainty in origin assignments, we used a bootstrapping approach by drawing 1000 samples of $b_{k}$ for each individual and estimated the dispersal kernel parameters using the distribution of $D_{k}$ from each bootstrap sample.

Previous isotope‐based assignment studies have found that stable hydrogen isotopes are able to provide geographic resolution of approximately 100 km (Hobson 2005). Therefore, we defined a long‐distance immigrant as an individual with $D_{k}\geq 100$ km. For each bootstrap sample, we classified individuals as long‐distance immigrants ($z_{k}$ = 1 = long‐distance immigrant, $z_{k}$ = 0 = short‐distance immigrant) and estimated the immigration rate (which can alternatively be interpreted as the probability of long‐distance immigration) for each plot as $\frac{\sum z_{k[p]}}{n_{p}}$, where $n_{p}$ is the number of individuals sampled on the plot.

### Correlates of long‐distance immigration

We used the results from the assignment model to test predictions about how local characteristics influence the probability of long‐distance immigration in each plot. Specifically, we used the estimated long‐distance dispersal status of each individual as the response variable in a logistic regression model that included habitat amount, patch quality, and Wood Thrush density as predictors. To test for the non‐linear effect of conspecific density predicted by the ‘social cues’ hypothesis, the models included both linear and quadratic density terms. We fit models to each of the 1000 bootstrap replicates and report the mean and 2.5/97.5% quantiles of the estimated slope coefficients (hereafter referred to as the 95% confidence intervals). Because each habitat selection hypothesis makes specific predictions about the sign of the relationship between predictors and probability of immigration, we also used the proportion of the slope estimates from each model that were greater than (or less than) 0 as a one‐tailed test of the significance of our estimates. All steps in the analysis were done in R version 3.3.1 (R Core Team 2016). Initially, we fit models that included all four predictors and a plot‐level random effect to account for pseudo‐replication. However, preliminary analyses indicated that, due to the small number of study plots, random effect variance terms were not estimable in all models and uncertainty in the estimated effects of each predictor was very large. To reduce model complexity and improve parameter estimability, we chose to fit separate models for each explanatory variable without plot‐level random effects. Due to the low correlations between predictor variables, these alternative approaches produced similar conclusions about the effects of each predictor (Appendix S1). To determine whether our results were sensitive to the 100 km threshold used to define long‐distance dispersal, we also conducted these analyses using 50 km and 150 km thresholds (Appendix S1).

## RESULTS

**Figure 3:**
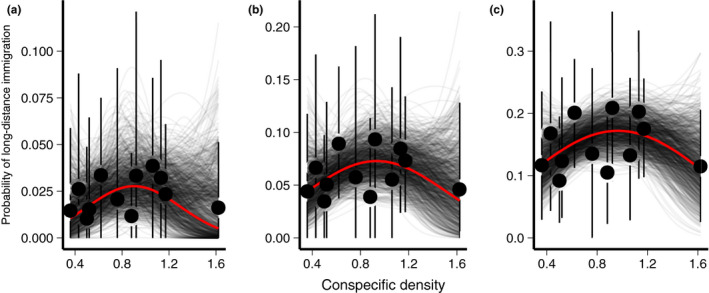
Effect of Wood Thrush density on the probability of long‐distance immigration under the (A) $\Gamma_{1}$, (B) $\Gamma_{2}$, and (C) $\Gamma_{3}$ prior distributions. Points and error bars show the estimated probability of long‐distance immigration and 95% confidence interval for each plot. Grey lines show the estimated effect of density on the probability of long‐distance immigration for each bootstrap sample and the red line shows the mean effect across all bootstraps.

Both the number of long‐distance immigrants (Table 1) and the probability of long‐distance dispersal (> 100 km) varied across the 12 study plots, ranging from 1% to 4% under the $\Gamma_{1}$prior, 3% – 9% under the $\Gamma_{2}$prior, 9% – 21% under the $\Gamma_{3}$prior. Overall, our results provide support for the predictions of the ‘social cues’ hypothesis. We found consistent evidence of a positive linear relationship and negative quadratic relationship between conspecific density and the probability of long‐distance immigration, indicating that the probability of long‐distance immigration was lowest in plots with very low or very high density (Fig. 3). The sign and magnitude of these effects were not sensitive to the choice of multiple‐predictor or single‐predictor models (Tables S1‐S6), though single‐predictor models had higher power to detect significant effects and are presented here. Under the most restrictive prior ($\Gamma_{1}$), the small number of observed long‐distance dispersal events resulted in highly uncertain parameter estimates (Pr(lineareffect>0)= 82%; Pr(quadraticeffect<0)=81%) but the signs of the parameters were consistent with predictions (estimated linear effect and 95% CI = 59.71, −48.12 to 254.48; estimated quadratic effect = −331.13, −1450.92 to 246.86). The less restrictive $\Gamma_{2}$prior resulted in more precise parameter estimates (linear effect = 30.13, −30.36 to 100.9; quadratic effect =−161.4, −580.88 to 143.22) and higher certainty in the signs of the effects (Pr(lineareffect>0)= 84%; Pr(quadraticeffect<0)=84%). Estimates using the least restrictive prior ($\Gamma_{3}$) were similar (linear effect = 23.02, −11.88 to 62.45; quadratic effect = −119.42, −340.54 to 60.45) but with even higher certainty in the signs of the effects (Pr(lineareffect>0)= 88%; Pr(quadraticeffect<0)=89%). Non‐linear effects of conspecific density were apparent even when the highest‐density plot was excluded from the analysis (Tables S7‐S9) and the observed patterns were consistent regardless of thresholds for defining long‐distance dispersal (50 km or 150 km; Tables S10–S15).

**Figure 4:**
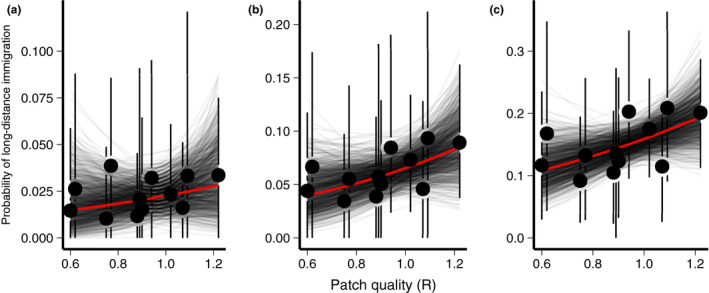
Effect of self‐recruitment rate (R) on the probability of long‐distance immigration under the (A) $\Gamma_{1}$, (B) $\Gamma_{2}$, and (C) $\Gamma_{3}$ prior distributions. Points and error bars show the estimated probability of long‐distance immigration and 95% confidence interval for each plot. Grey lines show the estimated effect of self‐recruitment rate on the probability of long‐distance immigration for each bootstrap sample and the red line shows the mean effect across all bootstraps.

The positive relationships between the probability of long‐distance immigration and self‐recruitment rate (i.e., patch quality R) were also consistent with the predictions of the ``social cues'' hypothesis (Fig. 4). Estimated effects of patch quality were similar across all three priors, ranging from 1.05 (95% CI = −2.46 to 4.62) under $\Gamma_{1}$ to 1.3 (−0.72 to 3.67) under $\Gamma_{2}$, and to 1.1 (−0.38 to 2.49) under $\Gamma_{3}$. As noted with the effects of conspecific density, confidence in the sign of the effect generally increased as the priors became less restrictive ($\Gamma_{1}$ Pr(slope>0)= 72%; $\Gamma_{2}$ Pr(slope>0)= 90; $\Gamma_{3}$ Pr(slope>0)= 94%). Again, these results were not sensitive to threshold used to define long‐distance dispersal (Tables S10‐S15) or to the choice of model structure (Tables S1‐S6).

In contrast, we found no evidence that habitat amount influenced the probability of long‐distance immigration (Fig. S1). Slope estimates were positive but close to zero under all three prior distributions ($\Gamma_{1}$: 0.2, −2.76 to 3.9; $\Gamma_{2}$: 0.05, −1.82 to 2.3; $\Gamma_{3}$: −0.1, −1.63 to 1.44). As result, the probability that the slope estimates were positive was close to 50% under all three priors ($\Gamma_{1}$: 54%; $\Gamma_{2}$: 50%; $\Gamma_{3}$: 45%).

**Figure 5:**
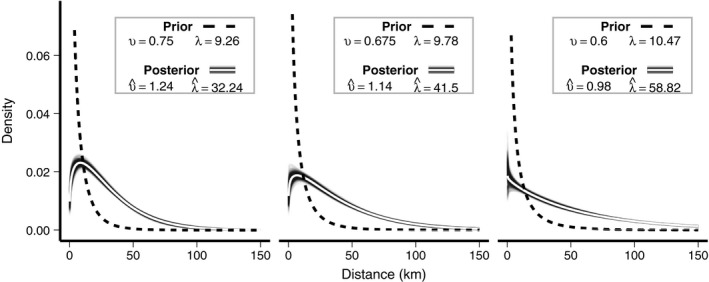
Prior and posterior dispersal kernels. Dashed lines show the three prior distributions used to define the dispersal kernel for juvenile Wood Thrush. All three have the same mean dispersal distance but imply different levels of long‐distance natal dispersal. Solid lines show the posterior mean dispersal kernels estimated from the assignment model. Compared to the three prior scenarios, the intrinsic marker data suggest more frequent long‐distance dispersal events.

Posterior estimates from our assignment model indicate that the mean natal dispersal distance of Wood Thrush in these populations was substantially farther than implied by the prior distributions used in the model (Fig. 5). Under the most restrictive prior ($\Gamma_{1}$), the estimated Weibull parameters were = $\hat{\upsilon }$1.24 (95% CI: 1.16–1.31) and = $\hat{\lambda }$32.24 (29.95–34.46), which implies a median dispersal distance of 24 km (95% CI: 22.32–25.63). Under the least restrictive prior ($\Gamma_{3}$), the estimated $\hat{\upsilon }$ parameter decreased to 0.98 (0.92–1.06) and the $\hat{\lambda }$ parameter increased to 58.82 (53.94–64.11), which corresponds to a median dispersal distance of 41 km (95% CI: 37.21–44.07).

## DISCUSSION

Despite a long history of theoretical investigations, tests of immigration theory are rare in wild animal populations due to the difficulty of quantifying long‐distance dispersal and identifying the geographic origin of immigrants. Here, we applied an innovative analytical framework to estimate dispersal distance using multiple intrinsic markers. We found evidence that long‐distance immigration of Wood Thrush was highest in high‐quality plots with moderate conspecific density, but unrelated to habitat amount.

These results are consistent with the hypothesis that dispersing individuals were precluded from settling in high‐density patches but used a combination of social and habitat cues to select the highest quality available patches. In our system, patch quality and conspecific density were positively correlated, but not perfectly so; some patches with high intrinsic quality had moderate‐to‐low density, possibly due to environmental and demographic stochasticity that occasionally reduced the number of breeding Wood Thrush relative to that predicted by patch quality. These mismatches between density and quality provide opportunities for dispersing individuals to settle in patches that they may otherwise be excluded from and in this way avoid being forced into the lowest quality plots. The ability to exploit these mismatches suggests that Wood Thrush are able to assess the quality of potential breeding patches, though our data did not allow us to determine the cues that they use to avoid low‐quality patches or identify high‐quality patches. Given that many organisms, including territorial songbirds, use the presence of conspecifics to select patches (e.g., Doligez *et al.,* 2003; Nocera *et al.,* 2006; Fletcher 2007), low conspecific density likely provided dispersing Wood Thrush with an efficient cue for avoiding low‐quality patches. It is also possible that individuals prospected for territories during the previous year, using information about the breeding performance of conspecifics to assess habitat quality and avoid low‐quality sites, a behavior that has been observed in several songbirds (Betts *et al.,* 2008; Kelly & Schmidt 2017). Conspecific density or performance alone, however, is not sufficient to explain our results because Wood Thrush were able to identify high‐quality patches even when density was low. Experimental investigations of songbird habitat selection have demonstrated that dispersing individuals use habitat features to augment conspecifics cues (e.g., Rushing *et al.,* 2015), suggesting that our results may be best explained by a combination of both conspecific and habitat cues. Regardless of the mechanisms, our results suggest the assumptions of ‘ideal’ habitat selection models may be appropriate in this and other avian populations, even for individuals with no previous knowledge of local breeding patches.

The ability of individuals to exploit mismatches between patch quality and conspecific density resulted in settlement patterns that were inconsistent with the predictions of the ‘ideal dominance’ model, which is the basis of source‐sink theory (Pulliam 1988) and often assumed to govern the dynamics of Wood Thrush and other migratory songbird populations (Robinson *et al.,* 1995; Brawn & Robinson 1996; Tittler *et al.,* 2006). Under source‐sink theory, source patches with high fecundity and adult fidelity produce an excess of recruits that are prevented from settling in their natal patch due to dominance of established individuals. These recruits are therefore forced to settle in low‐quality patches, resulting in a net movement of individuals from source to sink patches. Contrary to source‐sink theory, however, immigrants in our system were not forced into the lowest‐quality sink habitats but instead actively avoided these patches in favor of the highest‐quality patch available. As a result, immigration in some source habitats exceeded immigration in sinks. This subtle distinction between our results and classic source‐sink theory may have been missed by previous research because few studies of source‐sink dynamics have been able to separate immigrants from local recruits or directly quantify the relationship between immigration and habitat quality (but see Diffendorfer 1998).

The subtle distinction between our results and the predictions of source‐sink theory nevertheless have important implications for the dynamics of Wood Thrush and other bird populations that occur in fragmented landscapes. In any spatially structured population, the average per capita reproductive rate will be a direct function of the intrinsic quality of each patch and the distribution of individuals across patches. Per capita reproductive rate will be highest when individuals occupy only the highest quality patches and will decline as patches fill up, as the result of both within‐patch density dependence (i.e., crowding mechanism; Both & Visser 2000; Sillett *et al.,* 2004) and increasing number of individuals occupying lower‐quality patches (i.e., site‐dependent mechanisms; Ferrer & Donazar 1996; Rodenhouse *et al.,* 2003). Source‐sink theory predicts that per capita reproductive success will decline with increasing immigration rate. Our results suggest that immigrants are able to assess patch quality and conspecific density during settlement, thereby minimising the regulatory effects of both site‐dependent and crowding mechanisms. In this way, mismatches between density and patch quality, in combination with ideal habitat selection behavior by dispersing individuals, may weaken the strength of density dependence predicted by source‐sink theory, leading to higher per capita reproductive output of the entire population. These results also suggest that regional‐scale dispersal may buffer populations in high‐quality patches against demographic and environmental stochasticity, though the spatial resolution of isotopic signatures did not allow us to determine the relative contributions of local vs. regional dispersal to population dynamics in fragmented landscapes.

From a management perspective, our results underscore the importance of distinguishing between conspecific density and patch quality. Identifying high‐quality patches that potentially act as sources and distinguishing these patches from low‐quality sinks is a common management objective and our results substantiate previous research showing that conspecific density alone should not be used as a proxy for habitat quality given the possibility for mismatches between density and quality (Van Horne 1983). These mismatched sites may be particularly important for buffering populations from stochasticity by providing high‐quality territories to dispersing individuals. Our results suggest that a combination of conspecific density and stable isotope‐derived estimates of the probability of long‐distance immigration may serve as a sufficient proxy for habitat quality and thus for distinguishing between sources and sinks. Under this scenario, patches with high density and low immigration are likely to be high‐quality patches that may serve as sources due to their per capita and absolute productivity. Moderate density and high immigration may also indicate high‐quality patches that should be prioritised for protection whereas moderate density and moderate immigration may be indicative of low‐quality sink patches that are only maintained by immigration. Patches with both low density and low immigration are most likely very low quality and therefore should be the lowest priority for protection or management. Because ‘high’ vs. ‘low’ density and immigration rate can only be judged relative to other patches, these guidelines should be used only in cases where researchers are able to sample across a wide gradient both of metrics.

The dispersal kernels estimated from our data suggest that both the median natal dispersal distance and the probability of long‐distance natal dispersal in our study system were larger than predicted by the body mass scaling rule that we used as the basis for our prior distributions (Sutherland *et al.,* 2000). Relative to estimates of dispersal distance based on mark‐recapture data, our model may have overestimated the likelihood of long‐distance dispersal due to the low‐resolution of our intrinsic markers. Locations far from the study sites may have been associated with high likelihood of origin due to the inability of the isotope and wing chord data to discriminate between sites with approximately the same latitude. Future works based on higher‐resolution basemaps would also be useful for improving the the accuracy of assignments. However, the use of multiple markers, combined with the restrictive priors in our model, means that individuals that were classified as long‐distance immigrants had to have stable isotope and wing chord values that were both consistent with natal origins > 100 km from the study sites and inconsistent with natal origins < 100 km away. Thus, our model may actually be somewhat conservative about assigning long‐distance dispersal distances. In addition, our results are consistent with previous studies showing that even large‐scale mark‐recapture studies may substantially underestimate the frequency of long‐distance dispersal events (Tittler *et al.,* 2006).

The ability to detect long‐distance dispersal events for a large number of individuals after dispersal has already occurred is a key advantage of intrinsic markers. The method presented here advances the use of intrinsic markers by translating marker values into explicit estimates of dispersal distance with associated uncertainty. This method is easily extended to other intrinsic markers, including genetic markers or trace elements, or the inclusion of finer‐scale information about potential natal origins (e.g., Fournier *et al.,* 2017), opening the door for further study of the causes and consequences of long‐distance dispersal in many organisms. These tools will be increasingly important for advancing both the theory of dispersal and habitat selection. In addition, by providing a novel approach for identifying potential high‐quality source patches, this method can help prioritise which areas to protect in the face of habitat fragmentation and climate change.

## AUTHORSHIP

TBR, TSS, & PPM conceived of the study and oversaw data collection. CSR & JJV performed all analyses. CSR & TBR wrote the initial draft of the 1 manuscript and all authors contributed to subsequent revisions.

### Peer Review

The peer review history for this article is available at https://publons.com/publon/10.1111/ele.13729.

### OPEN RESEARCH BADGES

This article has earned Open Data and Open Materials badges. Data and materials are available at: https://doi.org/10.5061/dryad.qrfj6q5f5.

## Supporting information

Appendix S1

Appendix S2

Appendix S3

## Data Availability

All data and code associated with this manuscript are permanently archived and can be accessed from the Dryad Digital Data Repository (https://doi.org/10.5061/dryad.qrfj6q5f5).

## References

[ele13729-bib-0001] Avgar, T. , Betini, G.S. & Fryxell, J.M. (2020). Habitat selection patterns are density dependent under the ideal free distribution. J. Anim. Ecol., 89(12), 2777–2787. 32961607 10.1111/1365-2656.13352 PMC7756284

[ele13729-bib-0002] Betts, M.G. , Fahrig, L. , Hadley, A.S. , Halstead, K.E. , Bowman, J. , Robinson, W.D. *et al*. (2014). A species‐centered approach for uncovering generalities in organism responses to habitat loss and fragmentation. Ecography, 37, 517–527.

[ele13729-bib-0003] Betts, M.G. , Hadley, A.S. , Rodenhouse, N. & Nocera, J.J. (2008). Social information trumps vegetation structure in breeding‐site selection by a migrant songbird. Proceedings of the Royal Society B: Biological Sciences, 275, 2257–2263. 10.1098/rspb.2008.0217 PMC2603235 18559326

[ele13729-bib-0004] Bonte, D. , Dyck, H.V. , Bullock, J.M. , Coulon, A. , Delgado, M. , Gibbs, M. *et al*. (2012). Costs of dispersal. Biol. Rev., 87, 290–312. 21929715 10.1111/j.1469-185X.2011.00201.x

[ele13729-bib-0005] Both, C. & Visser, M.E. (2000). Breeding territory size affects fitness: An experimental study on competition at the individual level. J. Anim. Ecol., 69, 1021–1030.

[ele13729-bib-0006] Boughton, D.A. (2000). The dispersal system of a butterfly: A test of source‐sink theory suggests the intermediate‐scale hypothesis. Am. Nat., 156, 131–144. 10856197 10.1086/303380

[ele13729-bib-0007] Bowen, G.J. , Wassenaar, L.I. & Hobson, K.A. (2005). Global application of stable hydrogen and oxygen isotopes to wildlife forensics. Oecologia, 143, 337–348. 15726429 10.1007/s00442-004-1813-y

[ele13729-bib-0008] Bowler, D.E. & Benton, T.G. (2005). Causes and consequences of animal dispersal strategies: Relating individual behaviour to spatial dynamics. Biol. Rev., 80, 205–225. 15921049 10.1017/s1464793104006645

[ele13729-bib-0009] Brawn, J.D. & Robinson, S.K. (1996). Source‐sink population dynamics may complicate the interpretation of long‐ term census data. Ecology, 77, 3–12.

[ele13729-bib-0010] Chandler, R.B. , Royle, J.A. & King, D.I. (2011). Inference about density and temporary emigration in unmarked populations. Ecology, 92, 1429–1435. 21870617 10.1890/10-2433.1

[ele13729-bib-0011] Clobert, J. , Baguette, M. , Benton, T.G. & Bullock, J.M. (2012). Dispersal Ecology and Evolution. Oxford University Press, Oxford, UK.

[ele13729-bib-0012] Davis, J.M. & Stamps, J.A. (2004). The effect of natal experience on habitat preferences. Trends Ecol. Evol., 19, 411–416. 16701298 10.1016/j.tree.2004.04.006

[ele13729-bib-0013] Delignete‐Muller, M.L. & Dutang, C. (2015). Fitdistrplus}: An {R Package for Fitting Distributions. J. Stat. Softw., 64, 1–34.

[ele13729-bib-0014] DeSante, D.F. & Kaschube, D.R. (2009). The monitoring avian productivity and survivorship (MAPS) program 2004, 2005, and 2006 report. Bird Populations, 9, 86–169.

[ele13729-bib-0015] Diffendorfer, J.E. (1998). Testing models of source‐sink dynamics and balanced dispersal. Oikos, 81, 417–433.

[ele13729-bib-0016] Doligez, B. , Cadet, C. , Danchin, E. & Boulinier, T. (2003). When to use public information for breeding habitat selection? The role of environmental predictability and density dependence. Anim. Behav., 66, 973–988.

[ele13729-bib-0017] Ferrer, M. & Donazar, J.A. (1996). Density‐dependent fecundity by habitat heterogeneity in an increasing population of Spanish imperial eagles. Ecology, 77, 69–74.

[ele13729-bib-0018] Fletcher, R.J. (2007). Species interactions and population density mediate the use of social cues for habitat selection. J. Anim. Ecol., 76, 598–606. 17439476 10.1111/j.1365-2656.2007.01230.x

[ele13729-bib-0019] Fournier, A.M. , Drake, K.L. & Tozer, D.C. (2017). Using citizen science monitoring data in species distribution models to inform isotopic assignment of migratory connectivity in wetland birds. J. Avian Biol., 48, 1556–1562.

[ele13729-bib-0020] Fretwell, S.D. & Lucas, H.L. (1970). On territorial behavior and other factors influencing habitat distribution in Birds. I. Theoretical development. Acta Biotheoretica, 19, 16–36.

[ele13729-bib-0021] Fry, J.A. , Xian, G. , Jin, S.M. , Dewitz, J.A. , Homer, C.G. , Yang, L.M. *et al*. (2011). Completion of the 2006 National Land Cover Database for the conterminous United States. Photogramm. Eng. Remote Sensing, 77, 858–864.

[ele13729-bib-0022] Furrer, R.D. & Pasinelli, G. (2016). Empirical evidence for source–sink populations: A review on occurrence, assessments and implications. Biol. Rev., 91, 782–795. 26010659 10.1111/brv.12195

[ele13729-bib-0023] Greenwood, P.J. & Harvey, P.H. (1982). The natal and breeding dispersal of birds. Annu. Rev. Ecol. Syst., 13, 1–21.

[ele13729-bib-0024] Hanski, I. & Hanski, P. , in the D. of E. and S.I. (1999). Metapopulation Ecology. Oxford, UK: OUP Oxford.

[ele13729-bib-0025] Higgins, S.I. , Nathan, R. & Cain, M.L. (2003). Are long‐distance dispersal events in plants usually caused by nonstandard means of dispersal? Ecology, 84, 1945–1956.

[ele13729-bib-0026] Higgins, S.I. & Richardson, D.M. (1999). Predicting plant migration rates in a changing world: The role of long‐distance dispersal. Am. Nat., 153, 464–475. 29578791 10.1086/303193

[ele13729-bib-0027] Hobson, K.A. (2005). Using stable isotopes to trace long‐distance dispersal in birds and other taxa. Divers. Distrib., 11, 157–164.

[ele13729-bib-0028] Hobson, K.A. & Wassenaar, L.I. (1996). Linking breeding and wintering grounds of neotropical migrant songbirds using stable hydrogen isotopic analysis of feathers. Oecologia, 109, 142–148. 28307604 10.1007/s004420050068

[ele13729-bib-0029] Hobson, K.A. , Van Wilgenburg, S.L. , Wassenaar, L.I. & Larson, K. (2012). Linking hydrogen (2H) isotopes in feathers and precipitation: Sources of variance and consequences for assignment to isoscapes. PLoS One, 7, e35137. 22509393 10.1371/journal.pone.0035137 PMC3324428

[ele13729-bib-0030] Van Horne, B. (1983). Density as a misleading indicator of habitat quality. J. Wildl. Manag., 47, 893–901.

[ele13729-bib-0031] Jones, J. (2001). Habitat selection studies in avian ecology: A critical review. Auk, 118, 557–562.

[ele13729-bib-0032] Kelly, J.K. & Schmidt, K.A. (2017). Fledgling calls are a source of social information for conspecific, but not heterospecific, songbird territory selection. Ecosphere, 8, e01512.

[ele13729-bib-0033] Kokko, H. (1999). Competition for early arrival in migratory birds. J. Anim. Ecol., 68, 940–950.

[ele13729-bib-0034] Kot, M. , Lewis, M.A. & van den Driessche, P. (1996). Dispersal data and the spread of invading organisms. Ecology, 77, 2027–2042.

[ele13729-bib-0035] Laland, K.N. (2004). Social learning strategies. Animal Learning & Behavior, 32, 4–14. 10.3758/bf03196002 15161136

[ele13729-bib-0036] López‐Calderón, C. , Wilgenburg, S.L.V. , Roth, A.M. , Flaspohler, D.J. & Hobson, K.A. (2019). An evaluation of isotopic (2H) methods to provide estimates of avian breeding and natal dispersal. Ecosphere, 10, e02663.

[ele13729-bib-0037] Lowe, W.H. (2009). What drives long‐distance dispersal? A test of theoretical predictions. Ecology, 90, 1456–1462. 19569359 10.1890/08-1903.1

[ele13729-bib-0038] MacArthur, R.H. & Wilson, E.O. (2001). The Theory of Island Biogeography. Princeton, NJ: Princeton University Press.

[ele13729-bib-0039] Millon, A. , Lambin, X. , Devillard, S. & Schaub, M. (2019). Quantifying the contribution of immigration to population dynamics: A review of methods, evidence and perspectives in birds and mammals. Biol. Rev. 10.1111/brv.12549 31385391

[ele13729-bib-0040] Mitchell, M.S. , Lancia, R.A. & Gerwin, J.A. (2001). Using landscape‐level data to predict the distribution of birds on a managed forest: Effects of scale. Ecol. Appl., 11, 1692–1708.

[ele13729-bib-0041] Morris, D.W. (1987). Tests of density‐dependent habitat selection in a patchy environment. Ecol. Monogr., 57, 269–281.

[ele13729-bib-0042] Muller, K.L. , Stamps, J.A. , Krishnan, V.V. & Willits, N.H. (1997). The effects of conspecific attraction and habitat quality on habitat selection in territorial birds (Troglodytes aedon). Am. Nat., 150, 650–661. 18811306 10.1086/286087

[ele13729-bib-0043] Muller‐Landau, H.C. , Levin, S.A. & Keymer, J.E. (2003). Theoretical perspectives on evolution of long‐distance dispersal and the example of specialized pests. Ecology, 84, 1957–1967.

[ele13729-bib-0044] Nathan, R. , Perry, G. , Cronin, J.T. , Strand, A.E. & Cain, M.L. (2003). Methods for estimating long‐distance dispersal. Oikos, 103, 261–273.

[ele13729-bib-0045] Nocera, J.J. , Forbes, G.J. & Giraldeau, L.‐A. (2006). Inadvertent social information in breeding site selection of natal dispersing birds. Proceedings of the Royal Society B: Biological Sciences, 273, 349–355. 10.1098/rspb.2005.3318 PMC1560037 16543178

[ele13729-bib-0046] Norris, D.R. & Stutchbury, B.J. (2001). Extraterritorial movements of a forest songbird in a fragmented landscape. Conserv. Biol., 15, 729–736.

[ele13729-bib-0047] Paradis, E. , Baillie, S.R. , Sutherland, W.J. & Gregory, R.D. (1998). Patterns of natal and breeding dispersal in birds. J. Anim. Ecol., 67, 518–536.

[ele13729-bib-0048] Part, T. & Gustafsson, L. (1989). Breeding dispersal in the Collared Flycatcher (Ficedula albicollis): Possible causes and reproductive consequences. J. Anim. Ecol., 58, 305–320.

[ele13729-bib-0049] Petit, L.J. & Petit, D.R. (1996). Factors governing habitat selection by prothonotary warblers: field tests of the fretwell‐lucas models. Ecol. Monogr., 66, 367–387.

[ele13729-bib-0050] Pulliam, H.R. (1988). Sources, sinks, and population regulation. Am. Nat., 132, 652–661.

[ele13729-bib-0051] R Core Team (2016). R: A Language and Environment for Statistical Computing. R Foundation for Statistical Computing, Vienna, Austria.

[ele13729-bib-0052] Robinson, S.K. , Thompson, F.R. , Donovan, T.M. , Whitehead, D.R. & Faaborg, J. (1995). Regional forest fragmentation and the nesting success of migratory birds. Science, 267, 1987–1990. 17770113 10.1126/science.267.5206.1987

[ele13729-bib-0053] Rodenhouse, N.L. , Scott Sillett, T. , Doran, P.J. & Holmes, R.T. (2003). Multiple density–dependence mechanisms regulate a migratory bird population during the breeding season. Proc. R. Soc. Lond. B Biol. Sci., 270, 2105–2110. 10.1098/rspb.2003.2438 PMC1691488 14561272

[ele13729-bib-0054] Rousset, F. & Gandon, S. (2002). Evolution of the distribution of dispersal distance under distance‐dependent cost of dispersal. J. Evol. Biol., 15, 515–523.

[ele13729-bib-0055] Royle, J.A. & Rubenstein, D.R. (2004). The role of species abundance in determining breeding origins of migratory birds with stable isotopes. Ecol. Appl., 14, 1780–1788.

[ele13729-bib-0056] Rundel, C.W. , Wunder, M.B. , Alvarado, A.H. , Ruegg, K.C. , Harrigan, R. , Schuh, A. *et al*. (2013). Novel statistical methods for integrating genetic and stable isotope data to infer individual‐level migratory connectivity. Mol. Ecol., 22, 4163–4176. 23906339 10.1111/mec.12393

[ele13729-bib-0057] Runge, J.P. , Runge, M.C. , Nichols, J.D. , Stamps, A.E.J.A. & DeAngelis, E.D.L. (2006). The role of local populations within a landscape context: defining and classifying sources and sinks. Am. Nat., 167, 925–938. 16615034 10.1086/503531

[ele13729-bib-0058] Rushing, C.S. , Dudash, M.R. & Marra, P.P. (2015). Habitat features and long‐distance dispersal modify the use of social information by a long‐distance migratory bird. J. Anim. Ecol., 84, 1469–1479. 26061822 10.1111/1365-2656.12395

[ele13729-bib-0059] Rushing, C.S. , Hostetler, J.A. , Sillett, T.S. , Marra, P.P. , Rotenberg, J.A. & Ryder, T.B. (2017a). Spatial and temporal drivers of avian population dynamics across the annual cycle. Ecology, 98, 2837–2850. 28756623 10.1002/ecy.1967

[ele13729-bib-0060] Rushing, C.S. , Marra, P.P. & Studds, C.E. (2017b). Incorporating breeding abundance into spatial assignments on continuous surfaces. Ecol. Evol., 7, 3847–3855. 28616181 10.1002/ece3.2605 PMC5468143

[ele13729-bib-0061] Rushing, C.S. , Ryder, T.B. , Saracco, J.F. & Marra, P.P. (2014). Assessing migratory connectivity for a long‐distance migratory bird using multiple intrinsic markers. Ecol. Appl., 24, 445–456. 24834732 10.1890/13-1091.1

[ele13729-bib-0062] Sauer, J. , Hines, J. , Fallon, J. , Pardieck, K. , Ziolkowski, D. & Link, W. (2015). Breeding Bird Survey Summary and Analysis 1966–2013. Version 01.30. 2015. *USGS Patuxent Wildl. Res. Cent. Laurel MD. http://www. mbr‐pwrc. usgs. gov/bbs/bbs. html. Accessed*, 28.

[ele13729-bib-0063] Schmidt, K.A. , Johansson, J. & Betts, M.G. (2015). Information‐mediated allee effects in breeding habitat selection. Am. Nat., 186, E162–E171. 26655992 10.1086/683659

[ele13729-bib-0064] Sillett, T.S. , Rodenhouse, N.L. & Holmes, R.T. (2004). Experimentally reducing neighbor density affects reproduction and behavior of a migratory songbird. Ecology, 85, 2467–2477.

[ele13729-bib-0065] Smith, R.J. & Moore, F.R. (2005). Arrival timing and seasonal reproductive performance in a long‐distance migratory landbird. Behav. Ecol. Sociobiol., 57, 231–239.

[ele13729-bib-0066] Stamps, J.A. (1988). Conspecific attraction and aggregation in territorial species. Am. Nat., 131, 329–347.

[ele13729-bib-0067] Stamps, J.A. (2001). Habitat selection by dispersers: Integrating proximate and ultimate approaches. In Dispersal (eds Clobert, J. , Danchin, E. , Dhondt, A.A. , Nichols, J.D. , Wolff, J.O. ). Oxford University Press, Oxford, UK, pp. 230–242.

[ele13729-bib-0068] Stamps, J.A. , Krishnan, V.V. & Reid, M.L. (2005). Search costs and habitat selection by dispersers. Ecology, 86, 510–518.

[ele13729-bib-0069] Sutherland, G.D. , Harestad, A.S. , Price, K. & Lertzman, K.P. (2000). Scaling of natal dispersal distances in terrestrial birds and mammals. Conservation Ecology, 4, 16.

[ele13729-bib-0070] Szymkowiak, J. , Thomson, R.L. & Kuczyński, L. (2016). Wood warblers copy settlement decisions of poor quality conspecifics: Support for the tradeoff between the benefit of social information use and competition avoidance. Oikos, 125, 1561–1569.

[ele13729-bib-0071] Tittler, R. , Fahrig, L. & Villard, M.‐A. (2006). Evidence of large‐scale source‐sink dynamics and long‐distance dispersal among wood thrush populations. Ecology, 87, 3029–3036. 17249228 10.1890/0012-9658(2006)87[3029:eolsda]2.0.co;2

[ele13729-bib-0072] Trakhtenbrot, A. , Nathan, R. , Perry, G. & Richardson, D.M. (2005). The importance of long‐distance dispersal in biodiversity conservation. Divers. Distrib., 11, 173–181.

[ele13729-bib-0073] Valente, J.J. & Betts, M.G. (2019). Response to fragmentation by avian communities is mediated by species traits. Divers. Distrib., 25, 48–60.

