# Supplementary material for: Empirical tests of habitat selection theory reveal that conspecific density and patch quality, but not habitat amount, drive long‐distance immigration in a wild bird
Source: Ecol Lett. 2021 Mar 20;24(6):1167–77. doi: 10.1111/ele.13729 (PMC8251823; doi:10.1111/ele.13729)
Supplement: Supplementary file 1 — Appendix S1 [file ELE-24-1167-s002.pdf]

Appendix S1: Supplementary results

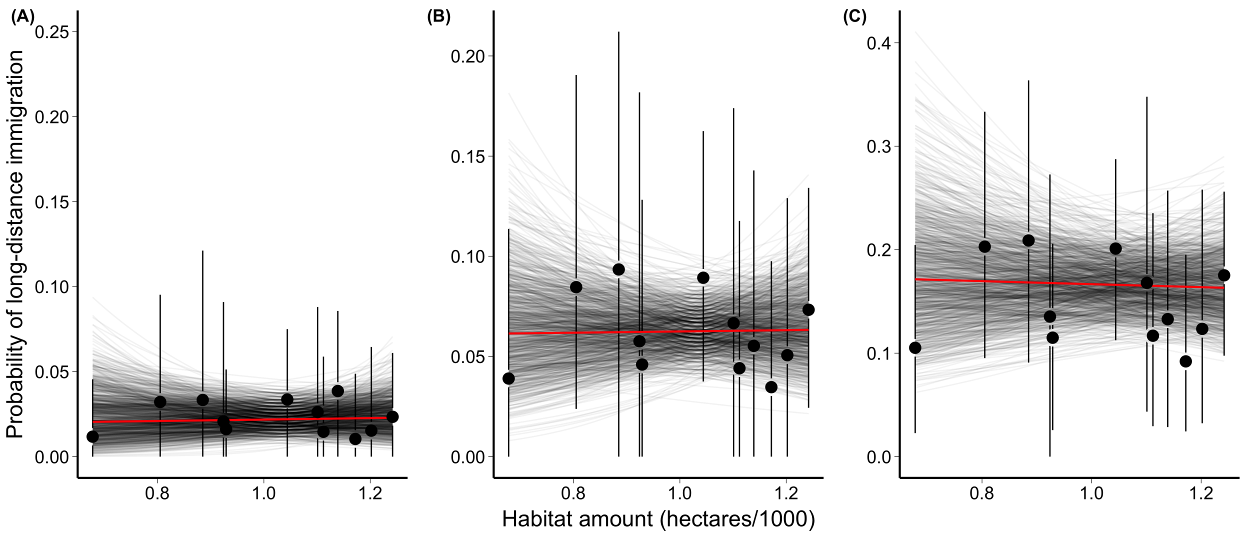

Figure S1: Effect of habitat amount on the probability of long-distance immigration under the (A) Γ_1, (B) Γ_2, and (C) Γ_3 prior distributions. Points and error bars show the estimated probability of long-distance immigration and 95% confidence interval for each plot. Grey lines show the estimated effect of habitat amount on the probability of long-distance immigration for each bootstrap sample and the red line shows the mean effect across all bootstraps.

Results of models with plot-level random effects

The following tables compare the results from the uni-predictor models presented in the paper to multi-predictor models that include plot-level random effects.

Table S1: Estimated effects of patch quality, conspecific density, habitat amount, and plot-level random effetcs on the probability of long-distance immigration, under the most restrictive dispersal prior (shape = 0.75, scale = 9.26). P is the proportion of bootstrapped effects that were greater than (or less than) zero.

Parameter	Mean effect	Lower 95% CI	Upper 95% CI	P
Habitat amount	0.18	-2.82	3.90	0.53
Patch quality	1.06	-4.38	6.00	0.64
Conspecific density	853.11	-62.03	280.81	0.79
Conspecific density (squared)	-3143.54	-1569.77	288.88	0.79

Table S2: Estimated effects of patch quality, conspecific density, habitat amount, and plot-level random effetcs on the probability of long-distance immigration, under the moderate dispersal prior (shape = 0.675, scale = 9.78). P is the proportion of bootstrapped effects that were greater than (or less than) zero.

Parameter	Mean effect	Lower 95% CI	Upper 95% CI	P
Habitat amount	0.05	-1.81	2.30	0.50
Patch quality	1.22	-0.99	3.63	0.86
Conspecific density	853.11	-43.44	96.96	0.76
Conspecific density (squared)	-127.60	-508.35	193.09	0.78

Table S3: Estimated effects of patch quality, conspecific density, habitat amount, and plot-level random effetcs on the probability of long-distance immigration, under the least restrictive dispersal prior (shape = 0.6, scale = 10.47). P is the proportion of bootstrapped effects that were greater than (or less than) zero.

Parameter	Mean effect	Lower 95% CI	Upper 95% CI	P
Habitat amount	0.00	-1.32	1.40	0.48
Patch quality	1.03	-0.51	2.51	0.92
Conspecific density	16.28	-21.36	58.54	0.78
Conspecific density (squared)	-90.53	-305.25	103.96	0.80

Results of multi-predictor models without random effects

The following tables compare the results from the uni-predictor models presented in the paper to multi-predictor models fit withoutplot-level random effects.

Table S4: Estimated effects of patch quality, conspecific density, and habitat amount on the probability of long-distance immigration, under the most restrictive dispersal prior (shape = 0.75, scale = 9.26). P is the proportion of bootstrapped effects that were greater than (or less than) zero.

Parameter	Mean effect	Lower 95% CI	Upper 95% CI	P
Habitat amount	2.58	-3.05	7.29	0.64
Patch quality	1.36	-4.60	6.48	0.64
Conspecific density	186.62	-78.80	503.00	0.78
Conspecific density (squared)	-1060.63	-3119.80	378.60	0.78

Table S5: Estimated effects of patch quality, conspecific density, and habitat amount on the probability of long-distance immigration, under the moderate dispersal prior (shape = 0.675, scale = 9.78). P is the proportion of bootstrapped effects that were greater than (or less than) zero.

Parameter	Mean effect	Lower 95% CI	Upper 95% CI	P
Habitat amount	0.29	-1.97	2.66	0.59
Patch quality	1.24	-1.03	3.64	0.87
Conspecific density	24.73	-45.07	119.86	0.75
Conspecific density (squared)	-139.33	-707.83	216.82	0.76

Table S6: Estimated effects of patch quality, conspecific density, and habitat amount on the probability of long-distance immigration, under the least restrictive dispersal prior (shape = 0.6, scale = 10.47). P is the proportion of bootstrapped effects that were greater than (or less than) zero.

Parameter	Mean effect	Lower 95% CI	Upper 95% CI	P
Habitat amount	0.19	-1.19	1.63	0.59
Patch quality	1.04	-0.51	2.50	0.92
Conspecific density	16.91	-22.64	63.60	0.78
Conspecific density (squared)	-93.86	-329.40	107.33	0.80

Effects of conspecific density without high-density plot

The following tables present results of the regression models from the paper, but without data from the high-density plot (plot 8 from table 1 in the main text). All models assumed a 100 km threshold for long-distance dispersal. Under the most restrictive prior (shape = 0.75), observed long-distance dispersal events were too rare to reliably estimate the non-linear effects of conspecific density (Table S7).

Table S7: Estimated effects of patch quality, conspecific density, and habitat amount on the probability of long-distance immigration, under the most restrictive dispersal prior (shape = 0.75, scale = 9.26) and without the highest density plot included. P is the proportion of bootstrapped effects that were greater than (or less than) zero.

Parameter	Mean effect	Lower 95% CI	Upper 95% CI	P
Habitat amount	0.62	-2.31	5.10	0.58
Patch quality	0.78	-2.98	4.78	0.67
Conspecific density	-3.79	-204.84	216.59	0.46
Conspecific density (squared)	-2.64	-1394.91	1234.43	0.48

Table S8: Estimated effects of patch quality, conspecific density, and habitat amount on the probability of long-distance immigration, under the moderate dispersal prior (shape = 0.675, scale = 9.78) and without the highest density plot included. P is the proportion of bootstrapped effects that were greater than (or less than) zero.

Parameter	Mean effect	Lower 95% CI	Upper 95% CI	P
Habitat amount	0.48	-1.64	2.85	0.64
Patch quality	1.34	-0.69	3.56	0.88
Conspecific density	19.52	-88.91	129.44	0.64
Conspecific density (squared)	-113.20	-805.81	569.09	0.62

Table S9: Estimated effects of patch quality, conspecific density, and habitat amount on the probability of long-distance immigration, under the least restrictive dispersal prior (shape = 0.6, scale = 10.47) and without the highest density plot included. P is the proportion of bootstrapped effects that were greater than (or less than) zero.

Parameter	Mean effect	Lower 95% CI	Upper 95% CI	P
Habitat amount	0.41	-0.94	1.92	0.70
Patch quality	1.20	-0.26	2.71	0.95
Conspecific density	12.31	-60.26	88.34	0.62
Conspecific density (squared)	-66.61	-537.07	374.38	0.61

Comparison of dispersal distance thresholds

The following tables compare the regression models presented in the paper, which use a 100 km threshold to define long-distance dispersal, to alternative 50 km and 150 km thresholds.

50 km threshold

Table S10: Estimated effects of patch quality, conspecific density, and habitat amount on the probability of long-distance immigration, under the most restrictive dispersal prior (shape = 0.75, scale = 9.26). P is the proportion of bootstrapped effects that were greater than (or less than) zero.

Parameter	Mean effect	Lower 95% CI	Upper 95% CI	P
Habitat amount	-0.24	-1.53	1.05	0.35
Patch quality	0.48	-0.71	1.82	0.77
Conspecific density	8.24	-26.19	44.05	0.67
Conspecific density (squared)	-40.27	-234.71	134.70	0.66

Table S11: Estimated effects of patch quality, conspecific density, and habitat amount on the probability of long-distance immigration, under the moderate dispersal prior (shape = 0.675, scale = 9.78). P is the proportion of bootstrapped effects that were greater than (or less than) zero.

Parameter	Mean effect	Lower 95% CI	Upper 95% CI	P
Habitat amount	-0.22	-1.34	0.91	0.35
Patch quality	0.59	-0.50	1.72	0.87
Conspecific density	10.01	-19.36	38.60	0.75
Conspecific density (squared)	-49.32	-206.59	98.55	0.73

Table S12: Estimated effects of patch quality, conspecific density, and habitat amount on the probability of long-distance immigration, under the least restrictive dispersal prior (shape = 0.6, scale = 10.47). P is the proportion of bootstrapped effects that were greater than (or less than) zero.

Parameter	Mean effect	Lower 95% CI	Upper 95% CI	P
Habitat amount	-0.34	-1.37	0.71	0.26
Patch quality	0.75	-0.16	1.74	0.94
Conspecific density	12.95	-11.27	38.88	0.84
Conspecific density (squared)	-64.04	-204.50	66.98	0.84

150 km threshold

Under the restrictive and moderate priors (shape = 0.75 and shape = 0.675), observed long-distance dispersal events were too rare to reliably estimate the non-linear effects of conspecific density (tables S10 & S11).

Table S13: Estimated effects of patch quality, conspecific density, and habitat amount on the probability of long-distance immigration, under the most restrictive dispersal prior (shape = 0.75, scale = 9.26). P is the proportion of bootstrapped effects that were greater than (or less than) zero.

Parameter	Mean effect	Lower 95% CI	Upper 95% CI	P
Habitat amount	29.00	-7.32	415.51	0.64
Patch quality	0.15	-18.95	132.69	0.68
Conspecific density	15675.73	-535.87	116552.19	0.87
Conspecific density (squared)	-94963.93	-662787.54	3483.21	0.87

Table S14: Estimated effects of patch quality, conspecific density, and habitat amount on the probability of long-distance immigration, under the moderate dispersal prior (shape = 0.675, scale = 9.78). P is the proportion of bootstrapped effects that were greater than (or less than) zero.

Parameter	Mean effect	Lower 95% CI	Upper 95% CI	P
Habitat amount	-0.04	-3.65	4.80	0.48
Patch quality	0.70	-2.94	5.91	0.76
Conspecific density	193.30	-63.52	333.70	0.78
Conspecific density (squared)	-1322.82	-2141.37	322.65	0.78

Table S15: Estimated effects of patch quality, conspecific density, and habitat amount on the probability of long-distance immigration, under the least restrictive dispersal prior (shape = 0.6, scale = 10.47). P is the proportion of bootstrapped effects that were greater than (or less than) zero.

Parameter	Mean effect	Lower 95% CI	Upper 95% CI	P
Habitat amount	-0.35	-2.98	1.94	0.40
Patch quality	0.99	-0.88	2.93	0.84
Conspecific density	26.00	-22.88	86.86	0.81
Conspecific density (squared)	-141.94	-503.31	125.48	0.81
