# Supplementary material for: Empirical tests of habitat selection theory reveal that conspecific density and patch quality, but not habitat amount, drive long‐distance immigration in a wild bird
Source: Ecol Lett. 2021 Mar 20;24(6):1167–77. doi: 10.1111/ele.13729 (PMC8251823; doi:10.1111/ele.13729)
Supplement: Supplementary file 2 — Appendix S2 [file ELE-24-1167-s001.pdf]

Appendix S2: Analysis code

2021-02-16

This vignette describes the data and analysis presented in Rushing et al. (*2021*) "Empirical tests of habitat selection theory reveal that conspecific density and patch quality, but not habitat amount, drive long-distance immigration in a wild bird" *Ecology Letters*.

Overview of assignment functions

The analysis uses two primary functions to assign individuals to natal origins. The function `disp_prob` (printed in full below) takes the intrinsic marker data for each individual and estimates the posterior probability that each potential site is an individual's natal origin. Given a matrix `coords` containing the longitude and latitude of each potential origin site and a numeric vector `site` containing the longitude and latitude of the sampling location, the function first estimates the geographic distance (in kilometers) between the sampling site and all other locations:

```
## Distance from focal site to all other cells
  d <- geosphere::distGeo(p1 = site, p2 = coords)/1000
```

Next, the function estimates the likelihood of dispersing to the study site from each potential natal site given the distances between the sites:

```
## Disperal likelihood
  disp_like <- dweibull(d, scale = Scale, shape = Shape)
```

Next, the disperal likelihoods are multiplied by the relative abundance of each cell and scaled to sum to 1. The resulting values are used as the prior probability of dispersing from each cell. Note that the object `abundance` should sum to 1.

```
## Disperal prior
  abun_like <- disp_like * abundance
  prior <- abun_like/sum(abun_like)
```

The marker values for each individual can be input as either a vector (1 marker) or matrix (> 1 marker). The dimensions of the data define the likelihood function used in the assignment (either normal or multi-variate normal). Users must also provide a vector or matrix `mu` containg the expected value(s) of the marker(s) at each potential origin:

```
## Assignment likelihood (1 marker)
  if(is.null(dim(data))){
    likelihood <- matrix(ncol = length(data), nrow = length(mu))
    for(j in 1:length(data)){
      likelihood[, j] <- dnorm(x = data[j], mean = mu, sd = Sigma)
    }
  }

  ## Assignment likelihood (>1 marker)
  if(!is.null(dim(data))){
    likelihood <- matrix(ncol = dim(data)[1], nrow = dim(mu)[1])
    for(j in 1:dim(data)[1]){
      likelihood[, j] <- apply(mu, 1,
                               function(x) mvtnorm::dmvnorm(x = data[j,],
                                                            mean = x,
                                                            sigma = Sigma))
    }
  }
```

Users should note that if only 1 marker is used, the `Sigma` argument in `disp_prob` is the *standard deviation* of the normal likelihood function (i.e., the standard deviation of marker values from individuals originating at the same site). If more than one marker is used, `Sigma` is the *variance-covariance* matrix of the multivariate normal likelihood.

Finally, the function multiplies the likeihoods by the prior and scales the posterior for each individual to sum to 1:

```
## Posterior probability
  posterior <- apply(likelihood, 2, function(x) x * prior)
  posterior <- apply(posterior, 2, function(x) x / sum(x))
```

The following code creates the `disp_prob` function:

```
disp_prob <- function(data, mu, Sigma, coords, abundance, site, Scale, Shape) {
  ## Distance from focal cell to all other cells
  d <- geosphere::distGeo(p1 = site, p2 = coords)/1000
  d[d == 0] <- 0.1 # Add small distance to avoid infinite likelihood

  ## Dispersal likelihood and abundance prior
  disp_like <- dweibull(d, scale = Scale, shape = Shape)
  abun_like <- disp_like * abundance
  prior <- abun_like/sum(abun_like)

  ## Assignment likelihood (1 marker)
  if(is.null(dim(data))){
```

```
    likelihood <- matrix(ncol = length(data), nrow = length(mu))
    for(j in 1:length(data)){
      likelihood[, j] <- dnorm(x = data[j], mean = mu, sd = Sigma)
    }
  }

  ## Assignment likelihood (>1 marker)
  if(!is.null(dim(data))){
    likelihood <- matrix(ncol = dim(data)[1], nrow = dim(mu)[1])
    for(j in 1:dim(data)[1]){
      likelihood[, j] <- apply(mu, 1,
                               function(x) mvtnorm::dmvnorm(x = data[j,],
                                                             mean = x,
                                                             sigma = Sigma))
    }
  }

  ## Posterior probability
  posterior <- apply(likelihood, 2, function(x) x * prior)
  posterior <- apply(posterior, 2, function(x) x/sum(x))

  return(posterior)
}
```

The other function, called `select_origin`, uses a bootstrapping approach to probablistically select origin cells for each individual based on the output posterior probabilities from `disp_prob`. The user can define the number of bootstrapped origins to select for each individual using the `nBoot` argument. The function then uses the `rmultinom()` function to randomly select a single origin cell (per bootstrap) for each individual, with probabilities determined by the cell posteriors. The output of this function is the bootstrapped dispersal distances for each individual and a vector indicating (for each bootstrap) whether the individual dispersed over a given distance. This distance is supplied as an argument to the function:

```
function(posterior, coords, site, distance = 100, nBoot = 1000){
  d <- geosphere::distGeo(p1 = site, p2 = coords)/1000
  l <- ifelse(d > distance, 1, 0)

  D <- matrix(NA, nrow = nBoot, ncol = dim(posterior)[2])
  z <- matrix(NA, nrow = nBoot, ncol = dim(posterior)[2])
  for(j in 1:nBoot){
    cell <- apply(posterior, 2,
```

```
                    function(x) which(rmultinom(n = 1, size = 1, prob = x) == 1))
    D[j,] <- d[cell]
    z[j,] <- l[cell]
  }

  return(list(z = z, D = D))
}
```

Data

Data necessary to replicate analyses from the manuscript can be loaded using the following code (after downloading from the Dryad repository):

```
mu <- readRDS(here::here("mu.rds"))
Sigma <- readRDS(here::here("sigma.rds"))
woth <- readRDS(here::here("woth.rds"))
coords <- readRDS(here::here("coords.rds"))
abundance <- readRDS(here::here("abundance.rds"))
plots <- readRDS(here::here("plots.rds"))
```

Our analysis used stable hydrogen isotope and morphological data from 506 second-year Wood Thrush breeding on 12 study plots in southern Indiana, USA. The `woth` data frame consists of $\delta^2 H$ values, wing chord length, and the breeding plot of each individual:

```
head(woth)
```

dd	wing	site
-49.19	105.0	mccr
-53.19	105.0	eabo
-51.57	105.0	spmi
-56.42	105.0	spmi
-63.68	103.5	spmi
-43.66	101.0	firs

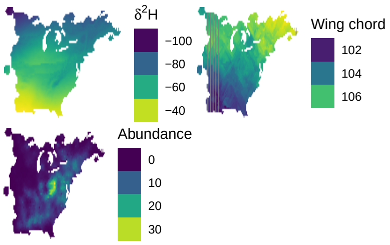

Figure 1: $\delta^2 H$, wing chord, and abundance basemaps

We assign individuals to potential natal sites using base maps that describe spatial variation in $\delta^2 H$, wing chord, and abundance.

The expected $\delta^2 H$ and wing chord values for each base map cell are stored in a matrix `mu` and relative abundance for each cell is stored in a vector `abundance`. Longitude and latitude of the cells are stored in a matrix `coords`.

In addition to the expected values for each cell, the assignment requires a variance-covariance matrix for $\delta^2 H$ and wing chord of bird

originating from the same sites. We estimate this variance-covariance matrix using $\delta^2 H$ and wing chord data from recaptured birds (i.e., known to have bred at the study sites in the previous year) on our plots and assume that within-site variation is constant across the range. The estimated matrix from these birds was:

```
Sigma
#>           [,1]      [,2]
#> [1,] 47.878198 -1.213328
#> [2,] -1.213328  9.201204
```

The last data object used for this analysis is a data frame containing the attributes of each study plot:

```
head(plots)
```

site	Latitude	Longitude	cell	R	ForCov	Density	n
ar07	39.04	-85.44	2086	1.02	1242	1.17	82
ar27	39.00	-85.38	2086	1.22	1044	0.62	80
ar41	38.93	-85.37	2153	1.09	885	0.92	33
ar58	38.84	-85.46	2153	0.94	805	1.13	42
eabo	38.79	-86.84	2149	0.75	1172	0.50	41
firs	38.87	-86.90	2149	0.62	1101	0.43	23

`cell` indicates which cell from the base maps each plot is located within, `R` is the estimated self-recruitment rate[1] for each plot, `ForCov` is the total amount of forest cover within a 2km-radius of each study plot, which we use a surrogate for habitat amount, and `Density` is the density of breeding Wood Thrush on each plot.

[1] Self-recruitment rate is the expected population growth rate λ of a population in the absence of immigration. In other words, the ability of a population to maintain itself through survival and recruitment

Assignment of juvenile Wood Thrush

Because our samples come from different plots (with different locations), we loop over each plot and use the `disp_prob` and `select_origin` functions to determine natal origins of individuals captured on that plot. Although we did not have an independent estimate of the dispersal kernel that describes the Wood Thrush natal dispersal distances, Sutherland et al. 2000 estimated a scaling function that predicts mean natal dispersal distance of birds based on body mass[2]. Breeding Wood Thrush are ~50g, suggesting a mean natal dispersal distance of ~5.6814971km. In the example below, we assume a Weibull dispersal kernel with scale = 5.6814971 and shape = 0.75. Under this kernel, long-distance dispersal should be more common than

[2] $36.4 \times M^{0.62}$

under the negative exponential kernel but should still be relatively uncommon (see below for further discussion of this assumption).

STABLE HYDROGEN ISOTOPES generally have an assignment resolution of ~100km and therefore we use this as the threshold to define long-distance dispersal in the `select_origin` function. Within the loop, we also estimate the probability of long-distance immigration for each plot as the number of long-distance immigrants divided by the total number of individuals. To capture uncertainty in this rate, we estimate immigration rate for each of the 1000 bootstrap iterations:

```
exp_D <- 36.4*(0.05^0.62)
Shape <- 0.75
for(i in 1:12){
  # Subset d2H and wing data for individuals on plot
  dat <- cbind(woth$dd[woth$site == plots$site[i]],
               woth$wing[woth$site == plots$site[i]])

  # Estimate posterior prob of origin for each indv
  posterior <- disp_prob(data = dat, mu, Sigma,
                                   coords, abundance,
                                   site = cbind(plots$Longitude[i], plots$Latitude[i]),
                                   Scale = exp_D/(log(2)^(1/Shape)), Shape = Shape)

  # Select nBoot origin cells for each indv based on posterior prob
  origins <- select_origin(posterior, coords,
                               site = cbind(plots$Longitude[i], plots$Latitude[i]),
                               distance = 100,
                               nBoot = 1000)

  # Estimate immigration rate for each bootstrap iteration
  ### 1 = long-distance immigrant, 0 = "local" so omega =
  ### mean(z)
  p <- apply(origins$z, 1, mean)

  ### Bind results
  if(i == 1){
    posteriors <- posterior # posterior prob
    Z <- origins$z          # dispersal status (1 = LDD)
    D <- origins$D
    P <- p                  # immigration rate estimates
  }else{
    posteriors <- cbind(posteriors, posterior)
```

```
    Z <- cbind(Z, origins$z)
    D <- cbind(D, origins$D)
    P <- cbind(P, p)
  }
}

save(P, Z, D, posteriors,
     file = here::here("results/dist100_shape0-75.RData"))
```

Correlates of long-distance immigration

One of the primary goals of our analysis was to determine whether patch-level characteristics like size or quality influence the rate of long-distance immigration in our study populations.We used the binary long-distance dispersal status (0 = local /short-distance immigrant, 1 = long-distance immigrant) from the assignment model as the response variable in a generalized linear model that included patch size, patch quality, and conspecific density as predictors. We fit this model for each of the 1000 bootstraps and report the mean and 95% credible intervals of each β coefficient:

```
## Load assignment results
load(here::here("results/dist100_shape0-75.RData"))

R <- rep(plots$R, table(woth$site))
R <- R - 1

FC <- rep(plots$ForCov/1000, table(woth$site))
FC <- FC - 1

Dn <- rep(plots$Density/10, table(woth$site))
Dn2 <- Dn^2

 ## Fit linear model to each bootstrap estimate of omega
  fc_mod  <- suppressWarnings(apply(Z, 1, function(x)
    coef(glm(x ~ FC, family = "binomial"))))

  save(fc_mod, file = here::here("results/dist100_shape0-75_fc_glm.RData"))

  ## Habitat quality
  R_mod  <- suppressWarnings(apply(Z, 1, function(x)
    coef(glm(x ~ R, family = "binomial"))))

  save(R_mod, file = here::here("results/dist100_shape0-75_R_glm.RData"))
```

```
## Density
D_mod  <- suppressWarnings(apply(Z, 1, function(x)
  coef(glm(x ~ Dn + I(Dn^2), family = "binomial"))))

save(D_mod, file = here::here("results/dist100_shape0-75_D_glm.RData"))
```
