# Supplementary material for: Empirical tests of habitat selection theory reveal that conspecific density and patch quality, but not habitat amount, drive long‐distance immigration in a wild bird
Source: Ecol Lett. 2021 Mar 20;24(6):1167–77. doi: 10.1111/ele.13729 (PMC8251823; doi:10.1111/ele.13729)
Supplement: Supplementary file 3 — Appendix S3 [file ELE-24-1167-s003.docx]

**Appendix S2 – Sampling and analysis methods for plot-specific density estimates**

We overlaid a 200 m square grid over each study plot and placed a point count station at each corner. The number of points per plot depended on plot size and ranged from 8 to 28 (mean = 18.8, sd = 4.33). Trained observers conducted 3 point counts per point in each of the 4 breeding seasons the plots were sampled (2011-2014). Point counts were conducted from 5:30 am to 10:30 am between 29 May and 16 July each year on mornings with suitable weather conditions (no rain and minimal wind). Repeated visits to sites were separated by approximately two weeks. Each count lasted 10 minutes during which time observers recorded all Wood Thrush seen or heard along with an estimated distance between the bird and the point count station. We only recorded birds detected within 100 m of the point, and thus the area of inference for each point is a 3.14 ha circle with a radius of 100 m (hereafter, a site).

We combined data from all 3 visits to a site within a year and used a generalized distance sampling model (Chandler et al. 2011) that allowed us to account for two sources of detection bias in our counts. The first is the probability an individual is detected given it is present in the 100 m radius circle (p_d_) which is modeled with the distance data. We modeled p_d_ using a half-normal detection function that varied by observer and scaled linearly with time of day and day of the year. The second is the probability an individual that uses the site is present in the 100 m radius circle during the 10-minute sample (p_p_, the inverse temporary emigration rate) which is estimated from the repeated visits to a site. We had no a priori reason to believe p_p_ would vary at the plot, site, or visit level, and thus we assumed it was constant across all surveys. Finally, we modeled abundance using a Poisson distribution where the expected value was a function of site, year, and a site-by-year interaction.

To estimate average plot-level densities, we first multiplied each expected abundance by 0.43, the model-estimated value of p_p_. This yields an estimate of the number of individuals expected to be present in a 100 m radius site at any moment in time. We then divided this by 3.14 ha, the area of one study site. This left us with 4 year-by-plot estimates of density which we averaged across years within a plot and present in Table 1 of the manuscript.
